# Trans-thoracic echocardiographic findings after the closure of ostium secundum atrial septal defect: A six-month follow-up study

**DOI:** 10.34172/jcvtr.025.32997

**Published:** 2025-03-18

**Authors:** Leila Bigdelu, Naser Nezhad Biglari, Yoones Ghaderi, Ali Azari, Maryam Emadzadeh, Mohsen Moohebati, Nadia Azadi, Vafa Baradaran Rahimi

**Affiliations:** ^1^Vascular and Endovascular Surgery Research Center, Mashhad University of Medical Sciences, Mashhad, Iran; ^2^Department of Cardiovascular Diseases, Faculty of Medicine, Mashhad University of Medical Sciences, Mashhad, Iran; ^3^Clinical Research Development Unit, Ghaem Hospital, Mashhad University of Medical Sciences, Mashhad, Iran

**Keywords:** Atrial septal defect, Echocardiography, Ventricular, Atrial, S’_TAPSE

## Abstract

**Introduction:**

Adults with an atrial septal defect (ASD) have the third most frequent congenital heart disease. We aimed to determine the echocardiography findings after the defect closure in patients with ostium secundum ASD.

**Methods:**

We included patients who underwent the closure of ostium secundum ASD using trans-catheter or surgical procedures. All patients were subjected to transthoracic echocardiography at admission, one month, and six months following successful closure. The remaining shunt were measured using contrast echocardiography and none of our patients had the remaining shunt.

**Results:**

We evaluated 28 patients with a mean age of 35.67±11.55 years. Twelve (42.85%) individuals had trans-catheter, and 16 (57.14%) patients had surgical closure of ASD. After ASD closure, the left ventricular (LV) ejection fraction (*P*=0.02), and LV end-diastolic diameter significantly increased while S’_TAPSE, right atrial (RA) area, RA volume, and pulmonary artery pressure (PAP) markedly diminished (*P*<0.001). During follow-up, RV size changes showed a significant decrease during one (17.93%) and six (25.78%) months (*P*<0.001 for both cases) and become normal following six months after the ASD closure. In addition, the RA/LA area ratio dropped by 24.31% during the first month and 33.17% after six months (*P*<0.001). In addition, the changes in measured echocardiographic parameters were not significantly different over time between the trans-catheter and surgical procedures. The decrease in S’_TAPSE was significantly greater in the surgical group than in trans-catheter closure.

**Conclusion:**

Closure of ostium secundum ASD dramatically decreased right cardiac chamber size and PAP while enhancing LV diameter and LV ejection fraction.

## Introduction

 Congenital heart diseases (CHD) are the leading cause of mortality in the first year of life. It is a subtype of heart disease that begins at birth and is often caused by aberrant development of normal fetal tissues or early development cessation. ^1,2^ The prevalence of this condition varies by area but is around 0.8 percent in total. Environmental and genetic factors have been implicated in the incidence and frequency of CHD. ^3,4^

 CHDs are classified as cyanotic or non-cyanotic. An atrial septal defect (ASD) is a non-cyanotic CHD that could present in four forms: ostium secundum ASD, ostium primum ASD, sinus venous ASD, and coronary sinus ASD. ^5^ ASD accounts for 10% of congenital cardiac abnormalities, and it is the third most frequent congenital heart disease in adults. ^6,7^ This defect has been linked to the development of pulmonary hypertension, congestive heart failure, atrial arrhythmias (mainly atrial fibrillation and atrial flutter), and decreased aerobic capacity, as well as various degrees of right heart volume overload and failure that may have significant detrimental consequences and in some cases, mortality. ^8,9^ Due to the possibility of such consequences, ASD is closed prophylactically throughout infancy and early adulthood. ^10^ Trans-catheter closure of ASD is one of the safest and most effective methods to reduce or eliminate atrial shunt. ASD closure via surgery is another method used in patients who are not candidates for catheter closure. ^11,12^

 Due to the significance of this disorder and the need to follow up patients after repair to determine the efficacy of treatment, we conducted the current study first to determine the transthoracic echocardiographic findings, especially right ventricular (RV) size after the defect closure in patients with ostium secundum ASD and compare them to the preoperative results. Second, we measured the course of changes and reduction of RV size and improvement of RV function one and six months after the closure of ASD.

## Materials and Methods

###  Study design and evaluation of outcome 

 The current cohort research included patients with ostium secundum ASD referred to the Cardiology Ward of Imam Reza and Ghaem Hospitals affiliated with the Mashhad University of Medical Sciences. The present study included 28 patients with ostium secundum ASD who had undergone surgical or trans-catheter closure. The indications for ostium secundum ASD closure were: clinical evidence of RV volume overload (pulmonary/systemic flow greater than 1.5 or RV enlargement), pulmonary artery pressure (PAP) lower than two-thirds of systemic arterial pressure, pulmonary vascular resistance lower than two-thirds of systemic resistance, or positive reoponse to pulmonary vasodilator testing. In transcathether ASD closure, self-centring devices need more than 5-7 mm of stable sufficient tissue to protect the stability of device. However, non-self-cnetering devices needs rims 50% larger than the device length. ^13,14^

 Transthoracic echocardiography (TTE) was performed using Siemens ACUSON SC2000 Ultrasound System with 4V1c Transducer (frequency bandwidth: 1.25-4.5 MHz). TTE was performed on all patients at admission, one and six months later, in the left lateral decubitus position. One echocardiography fellowship expert conducted all echocardiography following the current American Society of Echocardiography (ASE) guideline recommendations.

 In addition, the mid-level right ventricular size, tricuspid annular plane systolic excursion (S’_TAPSE), left ventricular ejection fraction (LVEF), left ventricular end-diastolic diameter (LVEDD), left ventricular end-systolic diameter (LVESD), pulmonary valve velocity time integral (PVVTI) and right ventricular outflow tract time integral (RVOTVTI), pulmonary artery pressure (PAP), left atrial (LA) area and volume, right atrial (RA) area and volume, and LA area/RA are were evaluated. After echocardiographic evaluations, the remaining shunt were measured using contrast echocardiography in all patients and none of our patients had the remaining shunt.

###  Inclusion and exclusion criteria

 All patients with ostium secundum ASD who had undergone surgical or trans-catheter closure were included in our study. Patients with the following conditions were excluded from this study: (1) prior right ventricular disease or dysfunction, (2) left ventricular myocardial infarction, (4) known pulmonary problems, (5) chronic obstructive pulmonary disease (COPD), (6) smoking, (7) significant left and right valvular heart disease, (8) moderate to severe left ventricular disease, (9) patients with arrhythmia such as atrial flutter, atrial fibrillation, left bundle block, atrioventricular (AV) block, and (10) pregnancy. Although AF is accompanied with advance defects, it leads to atrium enlargement that is a predisposing factor for persistent AF. Therefore, we excluded patients with AF to evaluate the pure effect of ASD closure.

###  Estimation of sample size

 The power (β) and the significance level (α) were set to 80% and 5%, respectively. Based on a previous study ^15^, according to the formula for comparing two means to obtain the difference of one unit in one variable (RV major dimension), the required sample size was 20. Since the estimated number of patients referred over two years was more than 20, we included all of them in our study.


$$N=\frac{{\left(Z_{1-\frac{\alpha }{2}}+Z_{1-\beta }\right)}^{2}\times \left(\sigma_{1}^{2}+\sigma_{2}^{2}\right)}{{\left(\mu 1-\mu 2\right)}^{2}}$$

Which $Z_{1-\frac{\alpha }{2}}=1.96,\;Z_{1-\beta }=0.84,\;\sigma_{1}=1.3,\;\sigma_{2}=1.1,\;\mu 1=6.9,\;and\;\mu 2=5.9$ . In this regard, a total of 28 patients were included in the present study.

###  Statistical analysis

 Data were analyzed using the SPSS version.22 statistical software (SPSS Inc., Chicago, Illinois). The parametric data were expressed as means ± SD, and non-parametric results were shown as a number with a percentage. The comparison between continuous variables was made using paired sample t-test. The P values (*P*) ≤ 0.05, 0.01, and 0.001 were considered statistically significant.

## Results

###  Study population

 A total of 28 patients participated in this study, of which five (17.86%) were male and 23 (82.14%) were female. The mean age of the participants was 35.67 ± 11.55 years. A total of 12 (42.85%) patients underwent trans-catheter closure, and 16 (57.15%) underwent ASD surgery.

###  Ventricular echocardiographic results

 Ventricularechocardiographic findings are shown in Table 1. Accordingly, the LVEDD significantly increased one month after treatment compared to the baseline (*P* < 0.001, Table 1). Additionally, after six months of ASD closure, LVEF (*P* = 0.02) and LVEDD (*P* < 0.001) notably enhanced compared to the baseline (Table 1). In contrast, S’_TAPSE markedly decreased one and six months after treatment compared to the baseline (*P* < 0.001 for all cases, Table 1).

**Table 1:** Ventricular echocardiographic findings at baseline and after one and six months of follow-up

**Characteristic**	**At admission**	**1-month follow-up**	**6-month follow-up**	**P1**	**P2**	**P3**
LVEF (%)	52.0.7 ± 3.91	53.03 ± 3.42	54.11 ± 3.48	0.19	0.02	0.01
LVEDD (mm)	4.06 ± 0.38	4.42 ± 0.36	4.55 ± 0.38	< 0.001	< 0.001	0.01
LVESD (mm)	2.74 ± 0.44	2.82 ± 0.26	2.85 ± 0.37	0.28	0.14	0.64
RV size (mm)	4.42 ± 0.7	3.59 ± 0.57	3.24 ± 0.44	< 0.001	< 0.001	< 0.001
S'_TAPSE	13.31 ± 3.21	9.53 ± 3.15	9.95 ± 2.35	< 0.001	< 0.001	0.28

LVEF: Left ventricular ejection fraction, LVEDD: Left Ventricular End-Diastolic Diameter, LVESD: Left Ventricular End-Systolic Diameter, TAPSE: Tricuspid annular plane systolic excursion; P1: P-value related to comparison of initial findings with 1-month follow-up using paired sample T-test, P2: P-value related to comparison of initial findings with 6-month follow-up using paired sample T-test, P3: P-value related to comparison of findings 1-month and 6-month follow-up using paired sample T-test.

**Figure 1:**
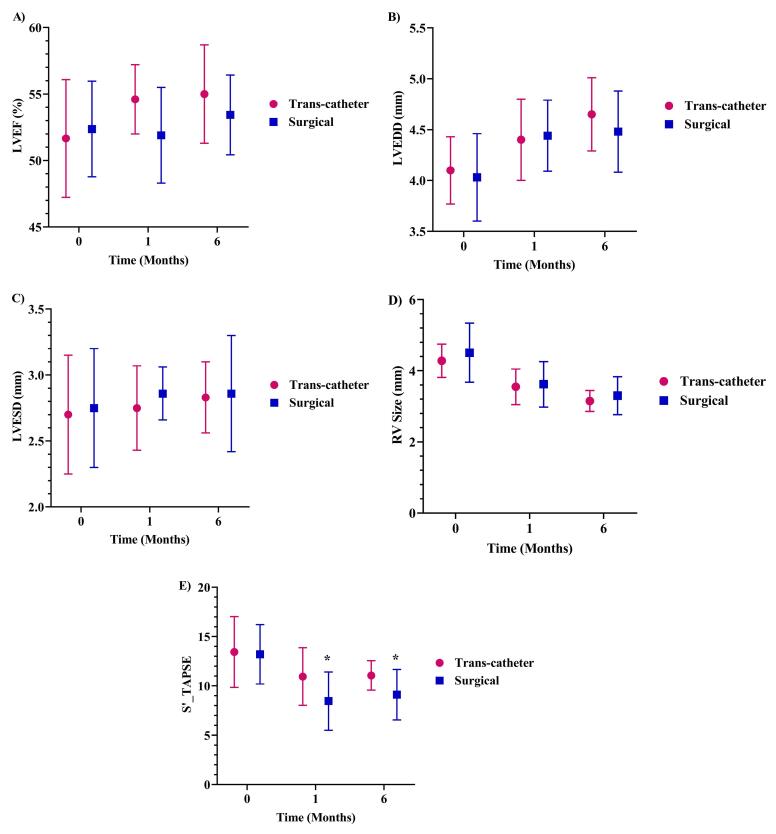
The trend of A) LVEF, B) LVEDD, C) LVESD, D) RV size, E) S’_TAPSE changes in the subjects according to the trans-catheter closure or surgery ASD repair method; ASD: atrial septal defect, LVEF: Left ventricular ejection fraction, LVEDD: Left Ventricular End-Diastolic Diameter, LVESD: Left Ventricular End-Systolic Diameter, TAPSE: Tricuspid annular plane systolic excursion; the comparison was performed using Independent T-test, * P < 0.05 between trans-catheter closure or surgery groups at the respected time.

 During follow-up, RV changes showed a significant decrease during one (17.93%) and six (25.78%) months of the study (decreasing from 4.42 ± 0.7 to 3.59 ± 0.57 after one month and 3.24 ± 0.44 after six months; *P* < 0.001 for both cases, Table 1). In addition, both trans-catheter and surgical ASD repair methods provided a significant decrement in the mid RV size during one (17% and 19.7%) and six (26% and 26.8%, respectively) months of follow-up (*P* < 0.001 for all cases, Figure 1).

 Our results also revealed that changes in LVEF, LVEDD, LVESD, and RV size over time were not significantly different between the two performed methods of trans-catheter closure and surgery (Figure 1). However, S’_TAPSE notably decreased in the surgical group than trans-catheter closure after one and six months of surgery (*P* < 0.05 for both cases, Figure 1).

###  Atrial echocardiographic results

**Figure 2:**
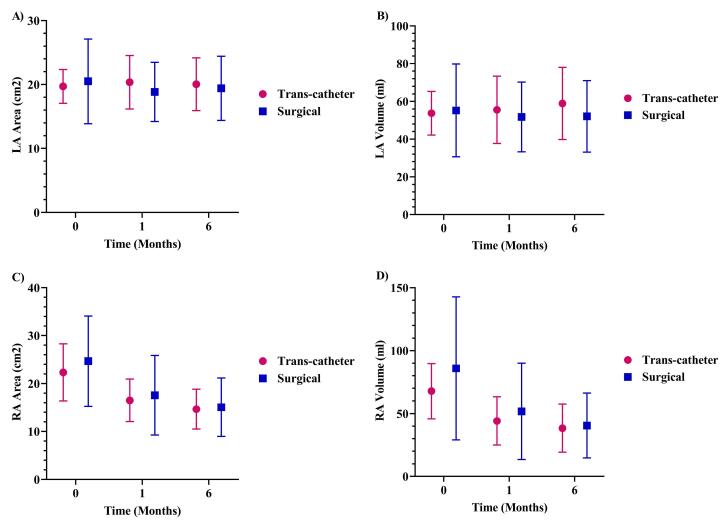
The trend of A) LA Area, B) LA volume, C) RA Area, D) RA volume changes in the subjects according to the trans-catheter closure or surgery ASD repair method at baseline and after one and six months of follow-up; RV: Right ventricular, LV: Left ventricular, ASD: atrial septal defect, the comparison was performed using Independent T-test.

 The LA area and volume did not change significantly in the studied patients during one and six months of follow-up (Table 2). The RA area at baseline was 23.67 ± 8.06 cm^2^, which decreased considerably to 17.10 ± 6.83 cm^2^ in the 1-month follow-up (*P* < 0.001), compared to 14.89 ± 5.24 cm^2^ in the 6-month follow-up, which had a significant reduction compared to the baseline (*P* < 0.001, Table 2). Additionally, the right-to-left atrial area ratio decreased by 24.31% during the first month and 33.17% during the six months of follow-up, which were statistically significant (*P* < 0.001, Table 2). Furthermore, the RA volume was remarkably mitigated in the first and sixth months compared to the first visit (*P* < 0.001, Table 2). Changes in the RA and LA volume and area over time were insignificant between the trans-catheter and surgical procedures (Figure 2).

**Table 2:** Atrial echocardiographic findings at baseline and after one and six months of follow-up

**Characteristic**	**At admission**	**1-month follow-up**	**6-month follow-up**	**P1**	**P2**	**P3**
LA area (cm^2^)	20.16 ± 5.24	19.50 ± 4.42	19.67 ± 4.58	0.38	0.51	0.59
LA volume (ml)	54.57 ± 19.76	53.35 ± 18.01	55.00 ± 18.97	0.64	0.89	0.40
RA area (cm^2^)	23.67 ± 8.06	17.10 ± 6.83	14.89 ± 5.24	*P* < 0.001	*P* < 0.001	0.002
RA volume (ml)	78.10 ± 45.53	48.50 ± 31.92	39.57 ± 22.79	*P* < 0.001	*P* < 0.001	0.004
RA area/LA are	1.18 ± 0.28	0.87 ± 0.24	0.75 ± 0.15	*P* < 0.001	*P* < 0.001	0.001

LA: Left atrium, RA: right atrium; P1: P-value related to comparison of initial findings with 1-month follow-up using paired sample T-test, P2: P-value related to comparison of initial findings with 6-month follow-up using paired sample T-test, P3: P-value related to comparison of findings 1-month and 6-month follow-up using paired sample T-test.

###  Echocardiographic findings of the pulmonary valve and artery

**Figure 3:**
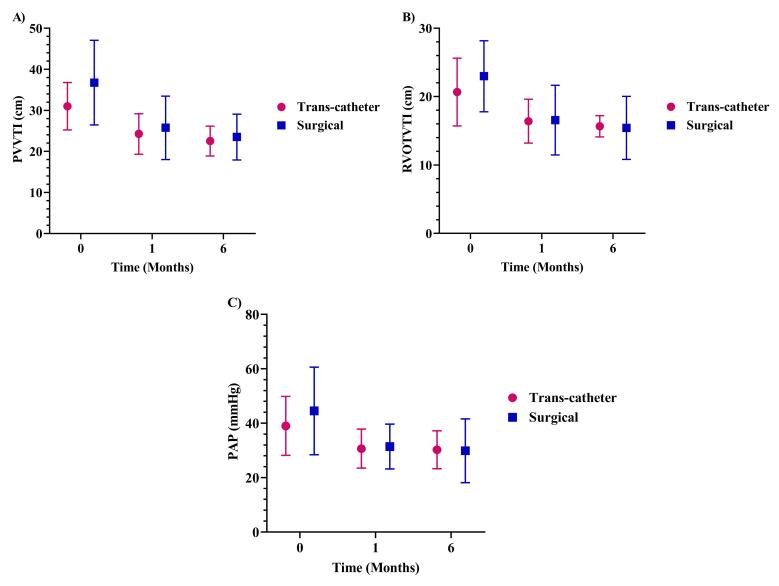
The trend of A) PVVTI, B) RVOTVTI, C) PAPchanges in the subjects according to the trans-catheter closure or surgery ASD repair method at baseline and after one and six months of follow-up; Pulmonary valve velocity time integral (PVVTI), right ventricle outflow tract velocity time integral (RVOTVTI), and pulmonary arterial pressure (PAP), ASD: atrial septal defect; thecomparison was performed using Independent T-test.

 The mean PVVTI was markedly alleviated by 24.09% (*P* < 0.001) during the first month and by 29.29% in the 6-month of follow-up (*P* < 0.001, Table 3). Moreover, the RVOTVTI and PAP strikingly reduced in the first and sixth months compared to the baseline (*P* < 0.001 for all cases, Table 3). In addition, the changes in PVVTI, RVOTVTI, and PAP were not significantly different over time between the trans-catheter and surgical procedures (Figure 3).

**Table 3:** Echocardiographic findings of the pulmonary valve and artery at baseline and during follow-up

**Characteristic**	**At admission**	**1-month follow-up**	**6-month follow-up**	**P1**	**P2**	**P3**
PVVTI (cm)	34.28 ± 9.01	25.10 ± 6.62	23.07 ± 4.79	< 0.001	< 0.001	0.44
RVOTVTI (cm)	21.98 ± 5.14	16.50 ± 4.30	15.53 ± 3.58	< 0.001	< 0.001	0.11
PAP (mmHg)	42.14 ± 14.14	31.10 ± 7.67	30.03 ± 9.81	*P* < 0.001	*P* < 0.001	0.36

Pulmonary valve velocity time integral (PVVTI), right ventricle outflow tract velocity time integral (RVOTVTI), and pulmonary arterial pressure (PAP); P1: P-value related to comparison of initial findings with 1-month follow-up using paired sample T-test, P2: P-value related to comparison of initial findings with 6-month follow-up using paired sample T-test, P3: P-value related to comparison of findings 1-month and 6-month follow-up using paired sample T-test.

## Discussion

 Trans-catheter closure is considered a safe and effective alternative to surgical repair for ASD closure. However, trans-catheter closure is not amenable for all patients, such as patients with complex ASD ^16^. The primary goal of this study was to assess the outcomes of ASD closure, especially RV size and its changes. In this context, echocardiographic findings were investigated through patient follow-ups before and after the surgery. According to the results of this study, RV size decreased considerably following one and six months after ASD closure. Moreover, the RV size was in the normal range following six months after the ASD closure. Comparing the changes in RV size among the two ASD repair procedures showed a similar and dramatic reduction over one and six months in both methods. The LA area and volume did not change considerably in this study. However, the RA area and volume, and PAP dropped significantly over the first and sixth months of follow-up after ASD closure.

 Trans-catheter ASD closure is now considered a standard clinical treatment. However, prolonged left-to-right shunting increases right ventricular volume and pulmonary vascular alterations in individuals with ASD, ultimately leading to increased RV pressure. Adults require more time to adjust to life following ASD closure than children.^1^ Long-term ASDs increase the left-to-right shunt, decreasing LV preload and cardiac output. Furthermore, by increasing the pulmonary artery pressure, this shunt increases the afterload of the right ventricle. ^17^ In patients with ASD, the volume of the right ventricular cavity will rise dramatically, pushing the interventricular septum wall towards the left ventricle, resulting in left ventricle compression. In this regard, we expect that the RV size will become normal after the ASD closure surgery, and if not, it may be due to the remaining left-to-right shunting. ^18^ Thus, as our findings showed, after ASD closure, the LV size grew significantly. In contrast, the right ventricular size decreased, enhancing LVEF in the study subjects. Interestingly, the RV size becomes normal six months after ASD closure. The reduction in RV diameter following ASD closure is consistent with Kort *et al.*^19^ and Veldtman *et al.*. ^20^ In addition, Supomo and coworkers also emphasized that the normalization of the RV function happens at six months after ASD closure. ^21^

 Cardiac magnetic resonance imaging (MRI) is the gold standard for evaluating right ventricular function. ^22^ Previous MRI examinations showed RV volume, diameter, and function improvement within six months of ASD closure. ^23^ Knepp and coworkers evaluated hemodynamic data such as RVEDD, LVEDD, and functional class of patients before and after ASD device closure. ^24^ They supported that right ventricular end-diastolic diameter (RVEDD) and LVEDD were both reduced, and the functional class improved. Behjati *et al.* evaluated the size of the left and right ventricles and their functional parameters in ASD patients before and after device closure. They found that ventricular size lowers while ventricular function improves after the treatment. ^25^

 Our results also revealed that the LA area and volume did not alter considerably one month and six months after ASD closure. In line with our results, Pascotto *et al.* found that the LA volume did not change significantly one month or six months following the intervention in 42 ASD patients in 2006. ^26^ Furthermore, Sigurdur S *et al.* performed another investigation in 2019 in which 16 ASD patients were assessed for echocardiographic results. According to the findings of this study, the volume of the LA did not change considerably during the one-year follow-up period. ^27^

 The current investigation found that the RA volume and area and PAP decreased significantly one and six months after the intervention compared to the beginning of the study. Several studies have looked into right atrial abnormalities in ASD patients. In this regard, Coomes* et al.* examined 80 ASD patients with trans-catheter closure of ASD in 2018. They determined that the volume of the RA reduced soon after ASD closure, and this tendency remained throughout the follow-up. ^28^ Similarly, Foo JS *et al.* demonstrated in 2018 that the right heart diameters and PAP dramatically decreased during one year of follow-up. ^29^ Pascotto *et al.* found that the volume of the RA reduced significantly 24 hours after surgery and during the 6-month follow-up period in ASD patients. ^26^

 We also found that S’_TAPSE markedly decreased one and six months after ASD closure. Interestingly, the decrease in S’_TAPSE was significantly greater in the surgical group than in trans-catheter closure. Similarly, Foo and coworkers suggested that trans-catheter closure of ASD remarkably preserved S’_TAPSE compared to surgical closure. ^29^ The decrease in S’_TAPSE in the surgical group may be due to the nature of the surgery and injury to the right ventricle. In fact, it may take more time to recover after surgery than trans-catheter closure. ^18,30,31^

 Finally, the present study had some limitations. First, we performed a single-center study with small sample size. Moreover, we did not perform MRI, the gold standard for evaluating RV function. On the other hand, the length of follow-up and assessment of various echocardiographic findings of patients are two of the study’s strengths. Therefore, we suggest that future research with larger sample sizes and longer follow-up times compare the echocardiographic outcomes of ASD surgical closure with the trans-catheter technique.

## Conclusion

 This study showed that closure of ostium secundum ASD significantly mitigated right cardiac chamber size and PAP while enhancing LV diameter and LV ejection fraction. Interestingly, the RV size becomes normal following six months after ASD closure. In this regard, abnormal RV size values after six months of ASD closure may be due to the remaining left-to-right shunting. Additionally, trans-catheter closure of ASD significantly preserved S’_TAPSE compared to surgical closure procedure.

## Competing Interests

 There is no conflict of interest.

## Ethical Approval

 This study was approved by the Ethics Committee of Mashhad University of Medical Sciences (approval code. IR.MUMS.MEDICAL.REC.1397.589). Furthermore, all participants received and signed written informed consent.
